# P-374. Long-term Effectiveness of Long-Acting Cabotegravir and Rilpivirine in an Infectious Disease Office-Based HIV Program

**DOI:** 10.1093/ofid/ofaf695.592

**Published:** 2026-01-11

**Authors:** Nikhil K Bhayani, Thomas K Sleweon, Erika M Young, Kent J Stock, Stacey E Baker, Quyen Luu, Jorge R Bernett, Brian S Metzger, Richard L Hengel, Richard C Prokesch, Kimberly A Couch, Christina J Weeks, Lucinda J Van Anglen

**Affiliations:** DFW Infectious Diseases, PLLC, Bedford, TX; Infectious Disease Specialists, Highland, Indiana; Infectious Disease Specialists, Highland, Indiana; Low Country Infectious Disease, Charleston, South Carolina; Infectious Disease Physicians, PA, Miami, Florida; Central Georgia Infectious Disease Associates, LLC, Macon, Georgia; Infectious Disease Doctors Medical Group, APC, Walnut Creek, California; Austin Infectious Disease Consultants, Austin, Texas; Atlanta ID Group, Atlanta, GA; Infectious Diseases Associates, PC, Riverday, Georgia; Healix Infusion Therapy, LLC, Sugar Land, Texas; Healix Infusion Therapy, LLC, Sugar Land, Texas; Healix Infusion Therapy, LLC, Sugar Land, Texas

## Abstract

**Background:**

Long-acting cabotegravir and rilpivirine (LA CAB/RPV) was approved in 2021 for treatment of HIV-1 to replace current antiretroviral therapy (ART) in those who are virologically suppressed with a viral load (VL) < 50 copies/mL. Challenges with patient access led to development of a standardized office-based LA CAB/RPV program in a network of Infectious Disease offices in the US. We reported initial success and patient adherence in 2024, and this study evaluates the long-term effectiveness of the LA CAB/RPV program.

**Table 1.**
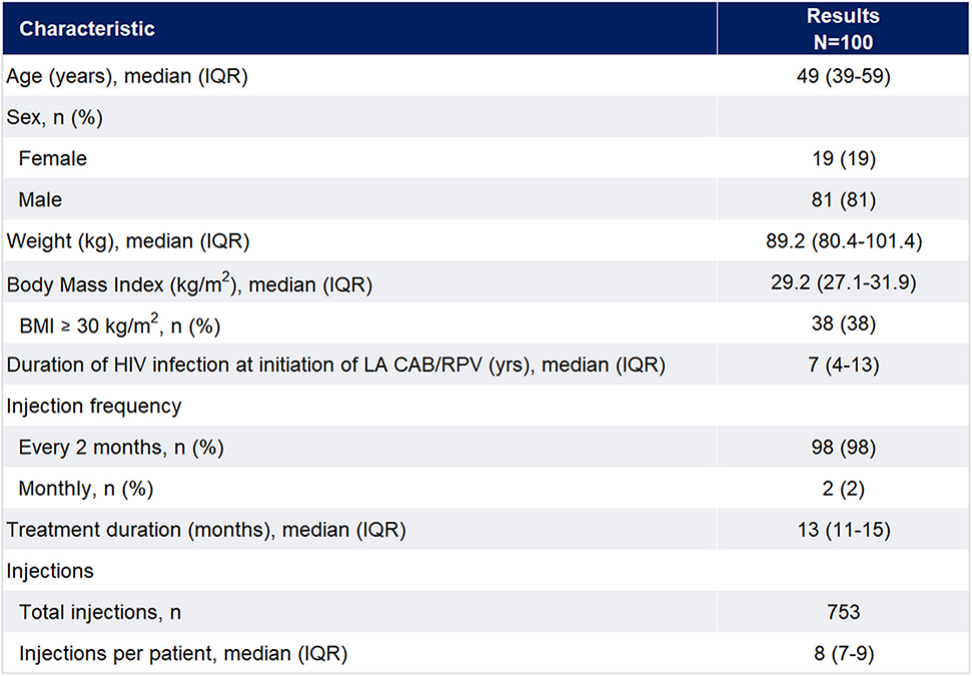
Baseline Demographics and Treatment Characteristics

**Figure 1.**
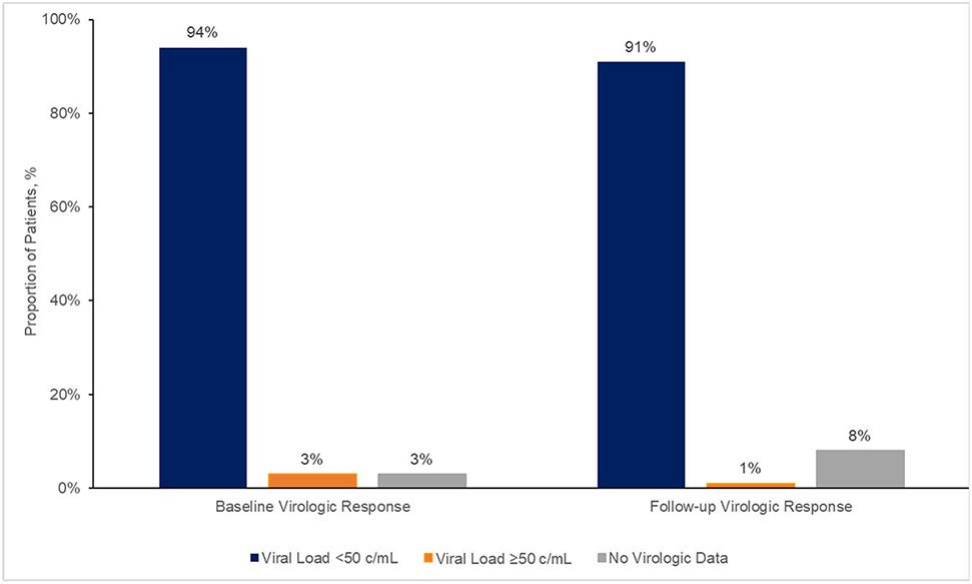
Virologic Outcomes at Baseline and Follow-Up

**Methods:**

The LA CAB/RPV program was initiated in July 2023 and the first 100 patients were followed through April 2025. Data collection included demographics, prior therapy, baseline and follow-up VL, adherence to schedule, adverse events, and reasons for discontinuation. Adherence was defined as LA CAB/RPV received within 7 days of target date.

**Table 2.**
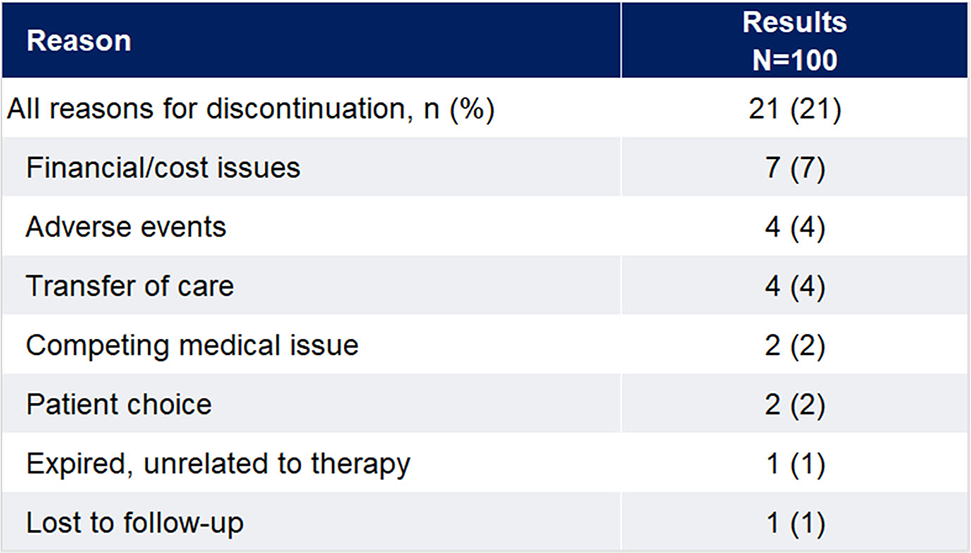
Reasons for discontinuation

**Results:**

100 pts from 20 sites were included. Patient and treatment characteristics are in Table 1. Median age was 49 years (IQR 39-59) with 81% male. Median BMI was 29.2 kg/m^2^ (IQR 27.1-31.9), with 38% ≥30 kg/m^2^. The majority (98%) received 2-month injections. The duration of therapy with LA CAB/RPV was 13 months (median, IQR 11-15), with 753 injections administered to date. Injections were administered on schedule in 94.8% of all doses. Virologic outcomes are noted in Figure 1. The baseline VL was 29 c/mL (median, IQR 20-54) and 94% were virologically suppressed at initiation of LA CAB/RPV. After a median of 8 doses (IQR 7-9) and for those with follow-up VL, 91 remained suppressed with a VL non-detectable (IQR 0-0). One had a VL of 84 copies/mL. Overall, 21 discontinued therapy, with reasons noted in Table 1. One third discontinued due to financial or payor issues. Non-serious adverse events occurred in 6%, with 4 resulting in discontinuation.

**Conclusion:**

An ID physician office based LA CAB/RPV program demonstrated durability in maintenance of virologic suppression with high injection adherence. These results suggest that LA CAB/RPV administered through a standardized ID office-based program is effective in managing HIV-1.

**Disclosures:**

Nikhil K. Bhayani, MD, FIDSA, Cumberland Pharmaceuticals: Advisor/Consultant|Melinta: Advisor/Consultant|Paratek Pharmaceuticals: Advisor/Consultant Brian S. Metzger, MD, MPH, Abbvie: Advisor/Consultant|Cumberland Pharmaceuticals: Advisor/Consultant|Ferring Pharmaceuticals: Advisor/Consultant Richard L. Hengel, MD, FIDSA, Gilead: Grant/Research Support Lucinda J. Van Anglen, PharmD, FIDSA, Cumberland Pharmaceuticals: Grant/Research Support|Ferring Pharmaceuticals: Grant/Research Support|Melinta Therapeutics: Grant/Research Support|Novartis: Grant/Research Support|Takeda Pharmaceuticals: Grant/Research Support

